# An Experimental Evaluation of Smart Sensors for Pedestrian Attribute Recognition Using Multi-Task Learning and Vision Language Models

**DOI:** 10.3390/s25061736

**Published:** 2025-03-11

**Authors:** Antonio Greco, Alessia Saggese, Carlo Sansone, Bruno Vento

**Affiliations:** 1 University of Salerno, 84084 Fisciano, SA, Italy; asaggese@unisa.it; 2 University of Naples Federico II, 80125 Napoli, NA, Italy; carlo.sansone@unina.it (C.S.); bruno.vento@unina.it (B.V.)

**Keywords:** pedestrian attribute recognition, contest, multi-task learning, vision language models

## Abstract

This paper presents the experimental evaluation and analyzes the results of the first edition of the pedestrian attribute recognition (PAR) contest, the international competition which focused on smart visual sensors based on multi-task computer vision methods for the recognition of binary and multi-class pedestrian attributes from images. The participant teams designed intelligent sensors based on vision-language models, transformers and convolutional neural networks that address the multi-label recognition problem leveraging task interdependencies to enhance model efficiency and effectiveness. Participants were provided with the MIVIA PAR Dataset, containing 105,244 annotated pedestrian images for training and validation, and their methods were evaluated on a private test set of over 20,000 images. In the paper, we analyze the smart visual sensors proposed by the participating teams, examining the results in terms of accuracy, standard deviation and confusion matrices and highlighting the correlations between design choices and performance. The results of this experimental evaluation, conducted in a challenging and realistic framework, suggest possible directions for future improvements in these smart sensors that are thoroughly discussed in the paper.

## 1. Introduction

The goal of a smart sensor for pedestrian attribute recognition is to simultaneously and automatically classify the characteristics of individuals, such as gender [1], clothing color, and the presence of hats or bags, from an image captured by a video surveillance camera [2]. This type of system is interesting for collecting statistics in retail or for person re-identification in forensics [3]. The challenge of implementing this smart sensor using an artificial vision system is related to the development of a neural network able to handle multiple classification tasks to identify each attribute [4,5]. This is particularly demanding when computational resources are limited, for instance, in several video surveillance applications that use embedded systems and cameras with limited computational capabilities [6].

To address the need to reduce the computational load of algorithms for multi-label classification problems, new neural models, specifically multi-task neural networks, have been developed in recent years [7]. These models are designed to solve multiple classification tasks simultaneously by sharing parts of their internal structure, such as the hidden layers, among various tasks [8]. Sharing weights reduces memory usage and processing time and leverages interdependencies between tasks, enhancing overall accuracy and generalization capabilities in pedestrian attribute recognition [9].

Building on these insights, the scientific community has developed several approaches based on multi-task neural networks for pedestrian attribute recognition, utilizing various architectures and input processing methods [2]. These algorithms can be categorized into those using global features (global-based approaches) extracted from the entire image and those detecting various parts of the human body (head, body, legs, etc.) and extracting features separately from these parts (part-based approaches).

One of the earliest global-based approaches is ACN [10], which employs a multi-branch model based on a pretrained version of CaffeNet, combined with multiple branches, one for each task. In order to exploit task interdependencies, the model is optimized using a Kullback–Leibler divergence-based loss function. DeepMAR [11] introduces a task hierarchy, hypothesizing that attributes like gender can influence feature representations for other attributes. Conversely, DeepSAR [11] treats each attribute independently, relying on the neural network to learn task interdependencies. Another global-based approach, MTCNN [12], employs multiple CNNs for feature extraction, a fully connected layer shared among these CNNs, and task-specific output layers. While global-based methods are simple, efficient, and robust for feature extraction, they can struggle with tasks requiring specific feature extraction from distinct parts of the sample (e.g., hat presence recognition).

Among part-based approaches, the authors in Poselet [13] classify attributes by dividing the sample into poselets, with a poselet being a particular part of the human pose under a given viewpoint. Features are thus extracted by the poselets and by the entire persons in a multi-scale fashion, and the classification is performed with multiple SVMs. RAD [14] uses a similar methodology: instead of using poselets, the image is partitioned into fixed-size overlapping subregions used for extracting features both individually and in multi-scale. A CNN for each task is thus employed. In [15], the OpenPifPaf algorithm has been used to extract the pedestrian skeleton; thus, each joint represents the basis for extracting local features from corresponding body parts. Although part-based features can enhance system performance, their effectiveness heavily depends on the accuracy of part localization. Inaccurate detection can lead to incorrect feature extraction, and additional time is required for part annotation and detection.

To overcome the limitations of both global-based and part-based methods while leveraging their strengths, recent approaches have introduced attention mechanisms in conjunction with multi-task networks. For example, the Multi-task Attention Networks (MTANET) model [16] uses attention mechanisms to process the entire pedestrian image and dynamically focus on key regions, such as the head for detecting a hat. This enables the model to automatically identify important areas specific to each task, eliminating the need for explicit part detection.

Building on this, transformer-based models, which inherently use attention mechanisms, have also been applied to the pedestrian attribute recognition problem. For instance, the PARFormer [17] model utilizes a multi-task transformer architecture. Recognizing the role of viewpoint in this problem, the authors treat the problem as two classification tasks, namely a viewpoint classifier (VC) and an attribute classifier (AC). These classifiers jointly guide the feature extraction, ensuring that both attribute and viewpoint data are taken into account.

However, transformer models, while promising, require large datasets to perform optimally. On smaller datasets, they are prone to overfitting, as they may capture noise and dataset-specific features, which limits their generalization abilities. This is why general-purpose architectures that utilize human-centered frameworks have also been developed. These frameworks address a variety of human-related perception tasks, such as pose estimation, pedestrian and skeleton detection, semantic part segmentation, attribute detection, and person re-identification. The key idea is to leverage the similarities between these tasks, allowing a single network to be dynamically queried during runtime based on specific questions. For example, in the UNIHCP model [18], users can ask whether the image contains a “female with a jacket”. The model then processes the entire image to infer the relevant attributes, requiring multiple queries to gather all attributes of an individual. Similarly, HULK [9] employs a transformer to convert RGB images into textual descriptions of pedestrian attributes. These models have the advantage of being trainable on vast datasets, such as HULK’s 30 million samples. However, they also come with high computational demands, both during training and inference. For example, training UNIHCP required 120 h using 88 NVIDIA V100 GPUs. These resource-intensive demands make real-time operation impractical.

In light of the growing interest in pedestrian attribute recognition, the PAR 2023 contest [19] was organized to evaluate these approaches in a realistic and challenging scenario, developing effective and efficient algorithms that can turn a standard video surveillance camera into a smart visual sensor for pedestrian attribute recognition. Unlike the recent UPAR challenge [20], PAR 2023 focused on multi-task learning methods, introducing various challenges to the pedestrian attribute recognition problem. Participants were asked to solve not only binary tasks, like in other competitions: instead, three binary tasks (gender, bag presence, hat presence) and two multi-class tasks (upper clothing color, lower clothing color) with 11 categories each had to be solved by the participants. This choice required the design of a multi-task loss function that balanced the learning between tasks, which can be unbalanced due to the different loss functions and task difficulty levels. The training set is composed of a mix of partially annotated samples from public datasets and unpublished samples, implying the need for learning techniques that handle missing labels or use a subset of the available samples; the participants were also encouraged to extend the dataset with more annotations and/or training images. Finally, the test set used for PAR 2023 consisted of unpublished images and was never made available to participants; this means that it was not possible to specialize the methods on the test set or use samples also present in the test set for training. This choice ensures a reliable evaluation of the smart sensors in real-world applications. In this realistic, challenging framework, it is possible to draw more targeted and robust scientific conclusions, identifying strengths and limitations that allow to adequately evaluate the capabilities of smart sensors for pedestrian attribute recognition in real conditions.

In this paper, we go into the details of the experimental evaluation carried out in the PAR 2023 contest, describing the methods proposed by the participating teams, carefully analyzing the results achieved by the teams and finding the correlations with the design choices. The discussion of the experimental results, based on the accuracy, the standard deviation and the confusion matrices, allows to draw motivated conclusions about the current state of multi-task pedestrian attribute recognition and possible future developments to improve the effectiveness of the methods.

## 2. Contest Description

In this section, we describe both the task and the dataset of the PAR 2023 contest [19]. The MIVIA PAR Dataset, firstly proposed in the contest, comprises a total of 105,244 images, partitioned into 93,082 for the training set and the remaining 12,162 for the validation set. Both training and validation sets have been made publicly available to the scientific community (https://mivia.unisa.it/par2023/ accessed on 10 March 2025). The organizers provide the participants with the training and validation sets, while the test set is private and never shared with the participants. The methods submitted by each participant team have, therefore, been evaluated by the organizers on the test set and the final ranking was established according to the criteria detailed in Section 4.

The following attributes were considered: color of clothing (both upper and lower), the gender, the presence of the bag and the presence of the hat. Note that such attributes are very important in several application domains, such as surveillance, forensic search, or retail analytics. They can help, for instance, in visually distinguishing people in case it is required to locate missing persons or in case law enforcement requires quickly filtering surveillance footage based on specific criteria to locate a suspect (i.e., the suspect is a man with a hat, wearing a white t-shirt). Furthermore, in retail analytics, understanding the demographic and the presence of bags can influence marketing strategies and store layouts.

In more detail, the following labels were considered:

- Color of Clothing: It can be black, blue, brown, gray, green, orange, pink, purple, red, white and yellow represented, respectively, by the labels [1, 2, 3, 4, 5, 6, 7, 8, 9, 10, 11]. Separate labels are provided for the upper and lower parts of the body.
- Gender: The values considered are male and female, represented by [0, 1], respectively.
- Bag: The presence or absence of a bag is indicated by [0, 1].
- Hat: The presence or absence of a hat is also indicated by [0, 1].

**Figure 1:**
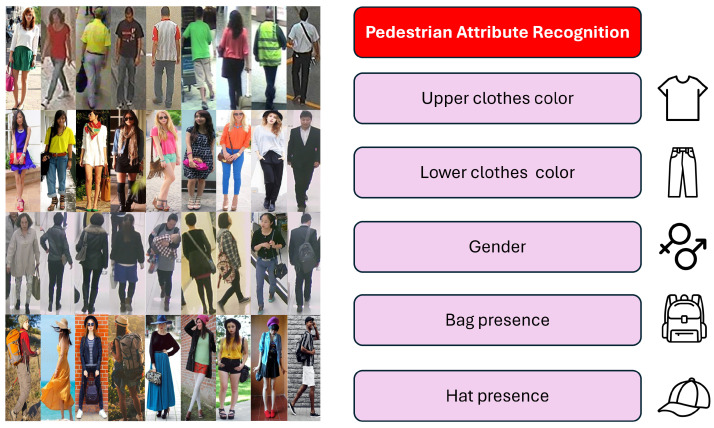
Pedestrian attribute recognition tasks and dataset examples with variations in terms of clothing color, gender, bag, hat, lighting, subject pose and image resolution.

Some samples in the MIVIA PAR Dataset have been sourced from existing datasets (e.g., PETA [21], RAP v2.0 [22]) and were manually annotated in case of missing attributes. Additionally, a significant portion of the dataset comprises private samples, for which the images were manually cropped and annotated with the relevant pedestrian attributes. Annotation was initially performed by a single annotator and subsequently verified by a second annotator. Due to the diverse conditions under which the images were collected, the dataset exhibits considerable heterogeneity in terms of image size, lighting, subject pose and distance from the camera. Examples of the images are reported in Figure 1.

Note that not all the attributes are available for all the sample images. Indeed, a value of −1 indicates the unavailability of a specific annotation. It causes an imbalance in the distribution of the samples in the training set. In total, the training set contains 26,076 fully annotated samples, while all samples in the validation set are fully annotated. In more detail, the training set includes the following:

- 35,847 samples with upper body color annotations,
- 60,759 with lower body color annotations,
- 85,142 with gender annotations,
- 65,684 with bag annotations,
- 78,271 with hat annotations.

Participants were thus encouraged to develop learning procedures that address this imbalance and also deal with missing labels.

**Figure 2:**
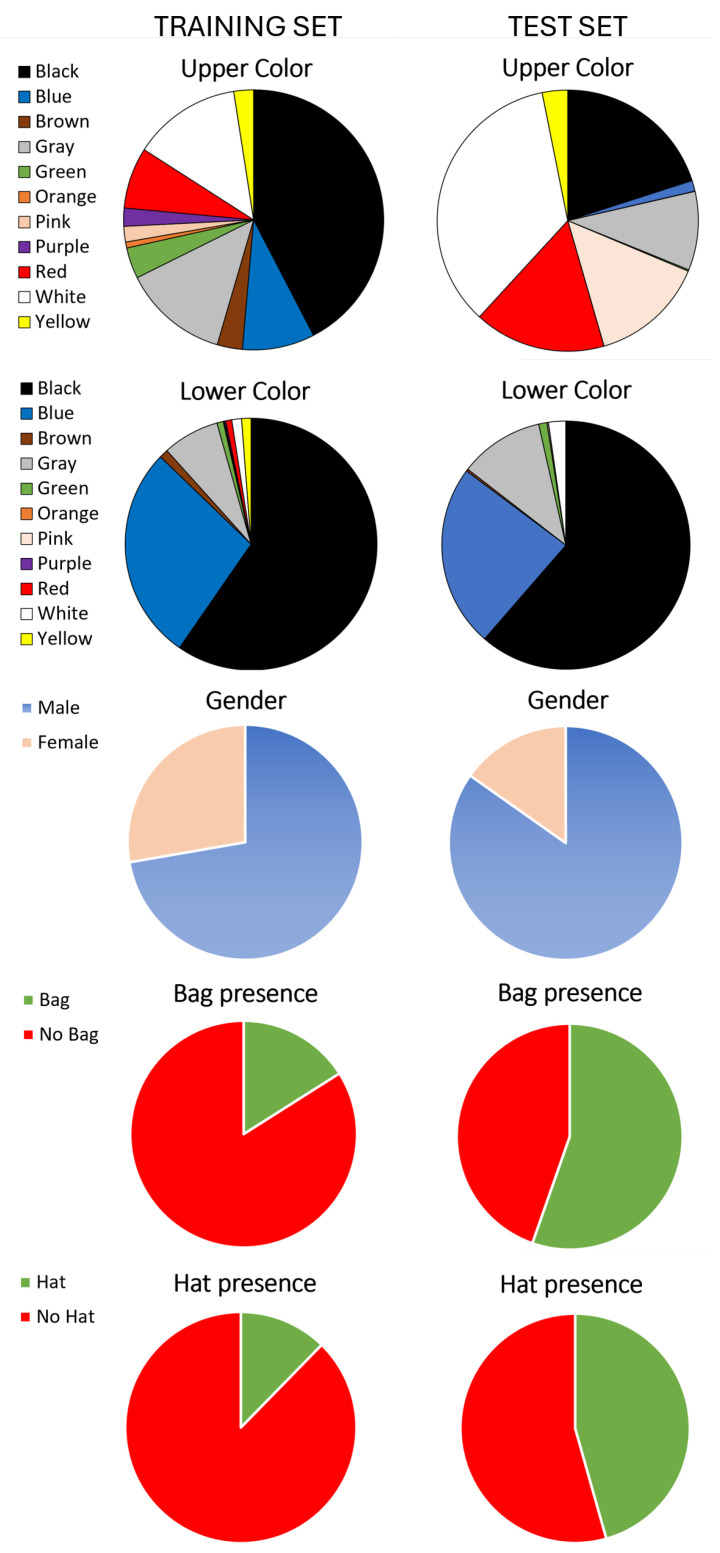
Distribution of the classes for the five tasks of interest in training and test set.

Note that an additional imbalance is in the class distribution, as shown in Figure 2: for the upper body color, the imbalance is less pronounced, with thousands of samples in categories, such as black (15,195), white (4828), gray (4714), blue (3225), red (2728), green (1381) and brown (1124). Conversely, the lower body color annotations show a notable concentration in black (36,228), blue (16,752) and gray (4427) samples. This imbalance reflects a real-world bias, as light-colored pants are less common. The training set predominantly consists of male (61,732), no bag (55,168) and no hat (68,629) samples.

The private test set used to evaluate the submitted methods consists of approximately 20,000 labeled images, captured using private surveillance cameras installed by the contest organizers to gather data from informed individuals with relevant attributes under a wide range of environmental conditions. The class distribution within the test set is unbalanced, especially in the classes representing clothing color and gender. Specifically, the class representing the color of the upper clothing assumes unbalanced values, with the white class predominating at 35%, followed by the black class at 20%. Other notable categories include red at 16%, pink at 14% and gray at 9%. As for the color of the lower clothing, further imbalance is observed: the black class is by far the most represented, accounting for 61%, followed by the blue class at 23%. In contrast, classes such as red and yellow are present with very few samples. The gender distribution is also unbalanced, with males representing 84% and females 16%. However, the bag presence class exhibits a more balanced distribution with no bag at 45% and bag at 55%. The hat class shows a similar trend with no hat at 55% and hat at 45%. Finally, regarding image size, the dataset spans a wide range of resolutions. The average image width is approximately 247 pixels, with an average height of 656 pixels. The minimum recorded width is 28 pixels, while the maximum width is 739 pixels. Similarly, the minimum height is 91 pixels and the maximum height is 1080 pixels. All the images in the dataset are in RGB format.

It is also worth noting that the participants were allowed to use additional data to train their network or to supplement samples with missing labels. In this case, they had to declare the additional data used or the additional labels so as to make the training reproducible.

## 3. Submitted Methods

Eleven teams from around the world have submitted official requests to participate in the contest, namely seven from Europe and four from Asia. In the end, five of them submitted a valid method to the contest: three from Europe and two from Asia.

In the following, the names of the team and the universities, the institutions or the company to which the teams belong to are reported: AWESOMEPAR (CASIA, Baidu—China), CODY (University of Oulu, Finland), HUSTNB (Huazhong University of Science and Technology—China), iROC-ULPGC (Universidad de Las Palmas de Gran Canaria, Universidad de La Laguna—Spain) [23], and SPARKY (University of Limoges, France). We describe the methods, summarized in Table 1, in the next paragraphs, reporting them in alphabetic order.

AWESOMEPAR. The team proposes an algorithm based on a Vision Transformer with Relation Exploration. For feature extraction, the Swin Transformer is used in combination with an Attribute and Contextual Feature Projection module. This module maps high-level features into attribute-specific representations while incorporating contextual information, allowing the model to capture relationships between attributes and different regions of the input image. The model processes inputs with a resolution of 224×224 pixels, which may slightly alter the person’s aspect ratio. The training data consist of a combination of the MIVIA PAR dataset, provided as part of the contest and the UPAR Challenge dataset, originating from another competition. Together, these datasets amount to 190,751 samples. Several data augmentation techniques were applied during training, including random flipping, cropping, scaling, translation, image erasure and Gaussian blur. Training was conducted using batches of 32 samples, with AdamW as the optimizer. The process began with a learning rate of $1\times 10^{-5}$ dynamically adjusted using a specific cosine scheduler.

CODY. This method is based, as is the solution presented by AWESOMEPAR, on a modified version of the Swin Transformer, specifically designed for the pedestrian attribute recognition task. Specifically, the model incorporates five distinct classification heads, each dedicated to a specific subtask within the PAR framework. The input to the model consists of images with a fixed resolution of 224×224 pixels, the same resolution used in the AWESOMEPAR’s solution. This ensures a square aspect ratio, which may slightly distort the person’s original proportions. Training was initially conducted on the contest-provided training set, followed by a fine-tuning phase based on performance metrics obtained from a validation set. The model was trained for a total of 300 epochs, using batches of 32 normalized images, with normalization performed using the mean and variance computed from the training set. For optimization, the Adam optimizer was employed with a learning rate of 0.0005. No regularization techniques were applied, keeping the weight decay parameter set to zero.

HUSTNB. This team adopts a different approach compared to the two previously described solutions. Instead of using a SwinT-based architecture, this team chose ResNet50 as the backbone, derived from the PLIP vision-language model, to learn a unique representation of each person. ResNet50 was pre-trained on the SYNTH-PEDES dataset, a large-scale person dataset with image–text pairs containing 312,321 identities, 4,791,711 images and 12,138,157 textual descriptions. To adapt the model for the PAR 2023 contest, five additional classification heads were integrated and the network was fine-tuned on the MIVIA PAR training set. The training process lasted for 50 epochs using cross-entropy loss and the Adam optimizer. The initial learning rate was set to 0.002, which was reduced to 0.0002 after 30 epochs. Several data augmentation techniques were applied during training, including random cropping, horizontal flipping and color normalization with batches of 128 samples.

iROC-ULPGC [23]. The authors approach the problem as a zero-shot VQA task. Specifically, they used the BLIP-2 model [24], a vision-language model pre-trained on approximately 130 million images from various datasets, including COCO, Visual Genome, CC12M, SBU and LAION400M. It is important to note that the model was not explicitly retrained for PAR. However, the authors demonstrate how the zero-shot generalization capabilities of BLIP-2 can be leveraged for new tasks or domains thanks to the learned semantics of words and images. To address the 2023 PAR contest task, the team adapted BLIP-2 by defining a set of contextual queries based on the validation set. These queries were designed through a process of prompt engineering. VQA responses, which are short and in natural language, are then mapped to the numerical labels required by the contest. For binary tasks, the mapping is straightforward, while color estimation tasks present a more complex scenario. The model may provide answers outside the eleven predefined color labels, such as multi-color or unannotated colors. To manage this, the team created additional mappings to handle such responses. For the upper clothing color recognition task, three queries were defined: “*What is the color of the person’s shirt?*”, “*Is the person wearing a jacket?*” and “*What color is the person’s jacket?*”. If the person is wearing a jacket, the third query is used to determine the color of the jacket; otherwise, the color of the shirt is returned. For the lower clothing color task, the query is as follows: “*What color are the person’s trousers?*”. For the gender classification task, the question is as follows: “*Is the person male or female?*”. For bag recognition, the selected query is as follows: “*Is the person wearing a bag?*”. Finally, for hat recognition, two questions were defined: “*Is the person wearing a hat?*” and “*Is the person wearing a cap?*”.

SPARKY. The method utilizes the integration of features from multiple backbones, including BeitV2, SwinT, EfficientNetV2, DaViT and CoAtNet. Each of these backbones contributes to extracting specific features based on their architecture and strengths. Specifically, the features extracted from BeitV2, SwinT and EfficientNetV2 are used for the recognition of bags and hats, tasks that require strong shape and detail extraction capabilities. DaViT, on the other hand, is added to handle gender classification, leveraging its architecture to capture subtle information that helps distinguish between male and female. Finally, all five backbones work together for clothing color recognition. Although the model employs five separate heads to tackle each task, all the backbones share weights, which helps reduce memory requirements. The model training process was performed using batches of eight 224×224 pixel images, similar to the setup used in CODY and AWESOMEPAR, extracted from the contest PAR 2023 contest’s dataset. During training, various data augmentation techniques were applied, including adjustments to the brightness and contrast of the images, allowing the model to adapt to different lighting conditions. Other techniques, such as horizontal flipping and blurring, were also used. Regarding optimization, the model was trained using the Adam optimizer and the training process lasted for a total of 40 epochs with a learning rate of $1\times 10^{-5}$.

## 4. Evaluation Metrics

In order to use a single metric to evaluate each method submitted to the contest, we use the mean accuracy across all considered tasks. In more detail, let $p_{i}$ represent the prediction for the *i*-th sample of the test set and $g_{i}$ the corresponding ground truth. The accuracy *A* is defined as the ratio of correct classifications to the total number of test samples *K*:

(1) $$A=\frac{\sum_{i=1}^{K}\left(p_{i}==g_{i}\right)}{K}$$

A higher accuracy score indicates greater effectiveness in recognizing the specific pedestrian attribute.

Note that each of the five pedestrian attributes has its own accuracy:

- $A_{u}$: Accuracy in recognizing the color of the clothes on the upper part of the body
- $A_{l}$: Accuracy in recognizing the color of the clothes on the lower part of the body
- $A_{g}$: Gender recognition accuracy
- $A_{b}$: Bag recognition accuracy
- $A_{h}$: Hat recognition accuracy

The contest ranking is determined by the mean accuracy (mA), which is the average performance across the five different pedestrian attributes:

(2) $$mA=\frac{A_{u}+A_{l}+A_{g}+A_{b}+A_{h}}{5}$$

In order to analyze the distribution of the performance among the tasks of interest, we also compute the standard deviation:

(3) $$St.Dev.=\sqrt{\frac{\sum_{k\in \{u,l,g,b,h\}}{\left(A_{k}-mA\right)}^{2}}{5}}$$

The lower the standard deviation achieved by an approach, the more balanced is its accuracy over the tasks.

Finally, to evaluate the processing speed of the methods, we compute the number of frames per second (FPS) that the approaches are able to process on a GPU NVIDIA Quadro RTX 8000. The higher the FPS, the faster the algorithm.

## 5. Results

The results of PAR 2023 are reported in Table 2. Together with the five teams participating in the contest, we also include in the table the baseline solution proposed in [19]; it consists of five single-task classifiers based on MobileNetv2, pretrained on ImageNet and fine-tuned on the images available in the training set of the MIVIA PAR Dataset.

The winner of the competition is iROC-ULPGC, which achieves the highest mA score of 0.921 and also the highest accuracy in four of the five tasks, demonstrating its robustness and reliability. The lowest value of the standard deviation (0.007) also demonstrates a certain stability of the classification accuracy, balanced over the various tasks of interest. These results confirm that employing a vision-language model, even if zero-shot but trained with hundreds of millions of images, is a viable solution also on the PAR task. Of course, this accuracy is paid with a low FPS (<0.5).

The runner-up is the method by the HUSTNB team, with an mA of 0.764 and a higher variability in its performance (standard deviation equal to 0.086) with respect to the winner team. Indeed, only $A_{h}$ and $A_{g}$ reach values higher than 0.84, with a very low $A_{b}$ (equal to 0.631). Although this is another vision-language model, in this case fine-tuned for the tasks of interest, we can note an impressive gap with the winner (0.921 vs. 0.764), with more than a 0.15-point difference. We can postulate that, in addition to the architectural differences between BLIP-2 and PLIP, using a large number of samples to train a generic vision-language model adapted zero-shot for the specific tasks of interest is more effective than using fewer samples for generic pre-training and fine-tuning with a few thousand samples on specific tasks. On the other hand, this solution is substantially faster than the winner (25 FPS).

The approach submitted by the SPARKY team, which is a complex multi-task classifier combining the features extracted with five different backbones, achieves an mA score of 0.753, which places it slightly behind HUSTNB. However, we can observe a higher variability in the accuracy among the tasks (0.126 vs. 0.086) from the standard deviation. In fact, the method exhibits high scores in $A_{l}$ (0.904) and $A_{g}$ (0.883) but a notably lower score in $A_{h}$ (0.577). We can suppose that such a complex ensemble would need more images than the ones available in the training set of the contest to achieve higher results, as well as a learning procedure that balances the performance among the tasks. Moreover, the complexity of the solution is also evident in the low processing speed (<0.5 FPS).

The other two methods, submitted by the CODY and AWESOMEPAR teams, were not able to outperform the baseline proposed in [19], which achieved an mA score of 0.709. While this score is lower than those of the top three methods, it serves as a useful relative benchmark for evaluating the results of the participants. Interestingly, the baseline showed a relatively balanced performance across the tasks, with scores ranging from 0.628 ($A_{u}$) to 0.832 ($A_{h}$), and a higher processing speed (150 FPS).

The multi-task approach submitted by the CODY team had an overall mA score of 0.636. It performed quite well in $A_{l}$ (0.743) and $A_{g}$ (0.848) but showed poor performance in $A_{b}$ (0.446) and $A_{h}$ (0.549). Looking at the difference in the distribution of samples in the training set and in the test set (shown in Figure 2) for bag recognition and hat recognition, we hypothesize that the approach is in overfitting on the training set; in fact, when the training and test set distributions are very similar (lower color and gender) or comparable (upper color), the accuracy is higher than in the other abovementioned cases. The CODY solution is able to process 10 FPS.

Finally, the AWESOMEPAR method achieved an impressive score of 0.936 in $A_{g}$ (being the best method on this specific task), but very low scores in $A_{u}$ (0.182) and $A_{l}$ (0.138), resulting in the lowest overall mA score of 0.557 (and the highest standard deviation of 0.330) among all the evaluated methods. Considering that a similar method performed admirably in the UPAR challenge, we can conclude that the PAR 2023 test set presents more challenging situations for this method and that the formulation of the color classification problem as a single multi-class task instead of a binary task for each color (as conducted in the UPAR challenge) creates more difficulties to this multi-task approach. Similarly to the CODY team, the SPARKY method can process 11 FPS.

## 6. Discussion

The experimental results of PAR 2023 show that methods based on vision-language models were able to achieve the first two places in the contest ranking.

Among the two approaches proposed by iROC-ULPGC and HUSTNB, the huge amount of training data (129 M images) used for the top-ranked method, not tailored to the problem of interest other than in the interpretation of the answers, has proven to be better compared to a model with relatively little pre-training data (less than 5 M images) fine-tuned on the tasks of interest. The third method in the ranking, submitted by SPARKY, obtained more or less the same results as HUSTNB with a model that was still very complex but trained with much less data, i.e., those available in the contest training set (around 100 K images). Therefore, the results suggest that using a vision-language model is an effective choice, but only if enormous quantities of training data are available; otherwise, a solution based only on image analysis can achieve comparable performance with much less data.

**Figure 3:**
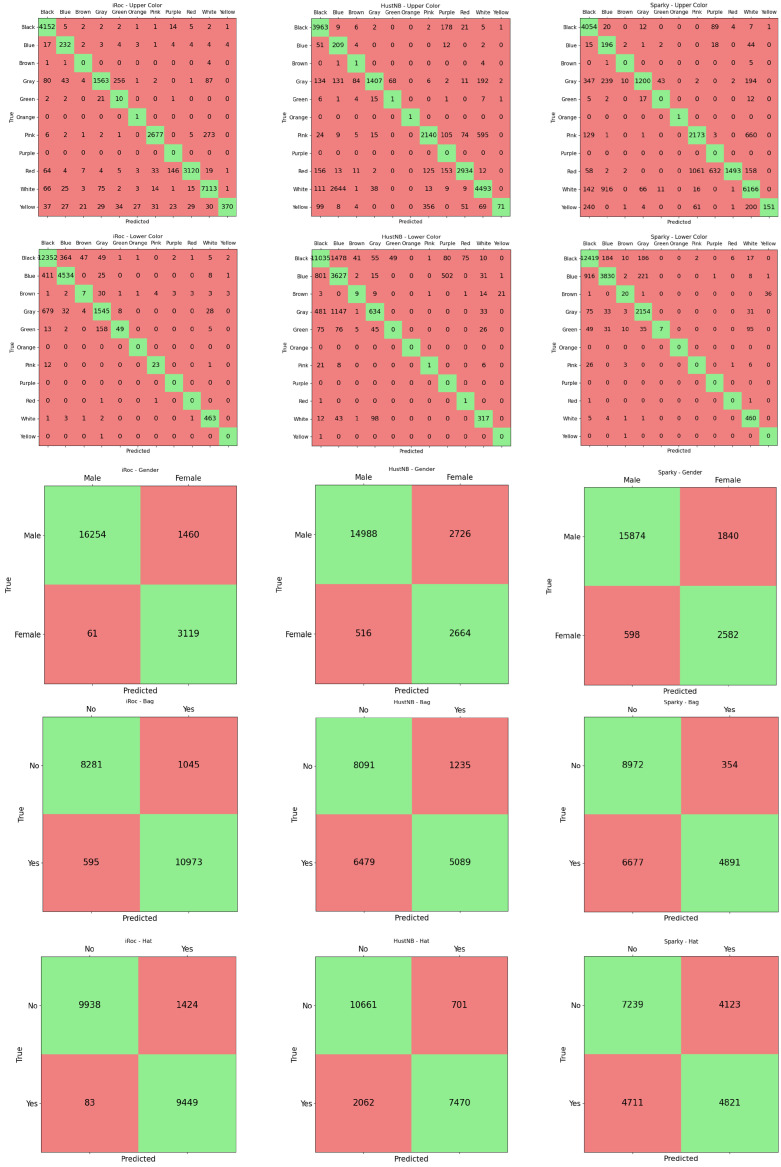
Confusion matrices of the three best teams, namely iROC-ULPGC, HUSTNB and SPARKY.

**Figure 4:**
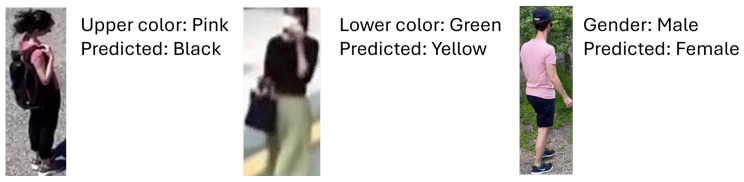
Examples of errors conducted by the method proposed by iROC-ULPGC team.

Analyzing the confusion matrices in Figure 3, we observe that the winner achieves more balanced accuracy than the other two contenders not only between the various tasks but also on the various classes of the specific tasks. This behavior is evident especially in the three binary tasks, namely gender, bag and hat recognition. On the two color classification tasks, we note that the errors in the iROC-ULPGC model are more explainable and regular. For example, on the upper color classification the confusion is mainly between similar colors (e.g., gray with white, black, blue, green; pink with white; red with purple) or with black (typical situation in images with shadow); on the lower color classification, the situation is even better, with errors mainly between black, blue and gray. The others make more varied errors, even when the accuracy on the task is comparable: the SPARKY team method achieves, on lower color classification, similar accuracy to the winner’s method (0.904 vs. 0.908) but, for example, confuses green with white. The errors are more related to the distribution of the samples in the training set than to the similarity between the classes involved in the misclassification. Qualitative examples of the errors conducted by the iROC-ULPGC model are reported in Figure 4; the errors are explainable by occlusions, similarity between the classes and clothes type. We can, therefore, note that the large amount of data used to train the iROC-ULPGC model also helps in stabilizing the results, with more understandable errors between the classes of interest; the other contenders have not considered these aspects of balancing between tasks and between classes of the same task in their training procedures.

In light of the peculiarities of the problem under consideration and the results of the contest, it is possible to hypothesize promising directions for improving pedestrian attribute recognition methods.

First, we observed that a vision-language model trained with huge amounts of data achieves impressive accuracy and regularity; it would be interesting to train a method based only on image analysis over as many samples annotated for the tasks of interest. Finding images of people should not be a big issue, while the availability of annotations for the various tasks is the real challenge; this problem could be overcome with training procedures designed for samples with missing labels or with knowledge distillation techniques guided by such foundation models as masters [25].

Second, we noted an imbalance in the accuracy on the various tasks, as evident from the analysis of the standard deviation. This can happen because the multi-task neural network learns weights for quite different tasks at the same time and, therefore, during the training procedure, may update the weights in favor of one or more tasks despite others. To solve this problem, it may be useful to apply methods that dynamically balance the multi-task loss, introducing a parameter that varies dynamically for each term in the cost function; the weights of the loss terms for the various tasks could be dynamically optimized to adapt to the learning evolution [26].

Third, from the analysis of the confusion matrices, we noticed that most of the methods achieve different accuracy on the various classes of interest in each specific task, as the imbalance in the dataset causes a bias in favor of the most represented classes. In this regard, almost all the proposed methods have used cross entropy, but in the presence of unbalanced datasets, it may be useful to choose loss functions appropriately designed for dealing with training set imbalance. These loss functions assign different weights to the samples for each class of interest; by appropriately configuring these weights, it could be possible to balance the learning with respect to the a priori distribution of the dataset and adapt the response of the neural network to the distribution expected in the real application [27].

Finally, even if we do not have evidence of this aspect from the results of the contest, in the literature some methods have improved PAR accuracy by focusing on the specific parts of the body affected by the attribute of interest (e.g., analysis in the head area to detect the presence of the hat). In this regard, multi-task attention networks (MTANs), with a CNN or Transformer-based backbone and task-specific branches that start with an attention mechanism, could learn how to exploit some local rather than global features to improve the accuracy on the specific task [28].

## 7. Conclusions

In conclusion, the PAR 2023 contest has provided valuable insights into the current state of smart sensors for multi-task pedestrian attribute recognition. Of course, the results should be considered in accordance with the operating conditions established in the competition and present in the test set, but the complexity and realism of the experimental framework allows us to make general observations. The results of the competition highlighted the effectiveness of smart sensors based on vision-language models trained with large datasets, suggesting that methods purely based on image analysis could benefit from large-scale annotated data. Addressing the challenge of annotation unavailability through advanced training techniques, such as handling missing labels and utilizing knowledge distillation, may be a winning strategy. The analysis also revealed imbalances in task accuracy. Dynamic balancing of multi-task loss functions discussed in Section 6 could mitigate this issue, ensuring more equitable weight updates across tasks. Furthermore, the performance disparities across different classes within each task indicate that using loss functions designed to handle dataset imbalance could improve overall accuracy. The findings from PAR 2023 emphasize the need for large-scale data, dynamic loss balancing, tailored loss functions, and attention mechanisms to advance pedestrian attribute recognition methods; however, the approaches proposed for the contest do not cover this entire range of variants. Future research should continue exploring these directions to improve the effectiveness and robustness of these approaches, which will allow the development of sensors that are even more efficient and sophisticated for pedestrian attribute recognition.

## Figures and Tables

**Table 1:** Overview of the methods used by the teams that attended the PAR 2023 contest.

Team	Method
AWESOMEPAR	SwinT with an ACFP module trained on the MIVIA PAR and UPAR datasets
CODY	SwinT modified with 5 classification heads trained on the MIVIA PAR dataset
HUSTNB	ResNet50 from PLIP trained on SYNTH-PEDES with classification heads fine tuned on the MIVIA PAR dataset
iROC-ULPGC [23]	Zero-shot VQA with BLIP-2 pre-trained on 130M images
SPARKY	BeitV2, SwinT, EfficientNetV2, DaViT and CoAtNet with 5 classification heads trained on the MIVIA PAR dataset

**Table 2:** Final results of PAR 2023 contest. The methods, including the baseline [19], are ordered on the basis of the mA, starting from the best method. The best results for each column are highlighted in bold.

Team	$A_{u}$	$A_{l}$	$A_{g}$	$A_{b}$	$A_{h}$	mA	St.Dev.	FPS
iROC-ULPGC [23]	**0.921**	**0.908**	0.927	**0.921**	**0.928**	**0.921**	**0.007**	<0.5
HUSTNB	0.728	0.748	0.845	0.631	0.868	0.764	0.086	25
SPARKY	0.739	0.904	0.883	0.663	0.577	0.753	0.126	<0.5
Baseline [19]	0.628	0.724	0.721	0.641	0.832	0.709	0.073	150
CODY	0.597	0.743	0.848	0.446	0.549	0.636	0.143	10
AWESOMEPAR	0.182	0.138	**0.936**	0.771	0.756	0.557	0.330	11

## Data Availability

The dataset is available under request at the following link: https://mivia.unisa.it/par2023/ (accessed on 10 March 2025).
